# Alabama Dental Association

**Published:** 1893-11

**Authors:** J. M. Walker


ARTICLE II.

### ALABAMA DENTAL ASSOCIATION.

BY MRS. J. M. WALKER.

*Concluded from page 273.*

### IN THE DISCUSSION OF PATHOLOGY.

Dr. L. G. Noel gave the history of a case of diseased antrum, with an abscess over the bicuspid roots, from constitutional vice; the bones were all affected, a portion of the septum nasi having been lost. After a long course of treatment she was dismissed as cured, but shortly after the patient fell down stairs and fractured the lower jaw. An interdental split was adjusted, but it is considered improbable that she will ever recover from the impairment of the osseous system, and exsection of a portion of the lower jaw may be necessary.

Dr. J. Y. Crawford, deprecated overtreatment of the antrum, being especially emphatic in the reiteration of *let it alone*, after drainage is secured and a condition of comparative comfort reached.

Dr. W. G. Browne gave the history of an interesting case, in which an accident, knocking out the deciduous teeth, resulted in diseased tissues, and the eruption of badly decayed, permanent incisors. These were early lost, the roots never being completely formed. At the age of fourteen years, Dr. Browne is about to insert a bridge, carrying the incisors.

Dr. George Eubank had a case in which, by a kick from a horse, a section of the lower jaw, carrying the six anterior teeth was broken at both ends, and driven back in the mouth half an inch. A jack screw was inserted between the bicuspids, driving them apart, till the broken piece was put in position, when it remained firmly in place without any splint.

Dr. Browne had a case in which the jaw was broken at the ramus. After this had healed the wisdom tooth erupted, pointing into the soft tissues of the cheek. Every effort was made to extract it, both by Dr. Browne and by several other dentists, but it could not be budged. Dr. Browne thought it would be necessary to saw it out.

Dr. C. N. Rosser, Atlanta, Ga., had a case with an abscess on the buccal side of a second bicuspid, which had been devitalized, and the root filled "by some other man." When lanced there was a free discharge of pus. After an absence from town of some six months on the part of Dr. Rosser, the young lady returned, the bicuspid having in the meantime been extracted by advice of her physician, and the abscess lanced again, but on the palatine surface. This opening had not healed, and on examination he found another opening an inch back, at the junction of the hard and soft palate. Water or medicine syringed into one opening came out from the other. There was considerable necrosed bone, a piece the size of the thumb nail, and four other smaller pieces being removed. It healed in about thirty days after the last operation, with a slight thickening of the membrane from front to rear.

Dr. R. C. Young spoke of the great value of pyrozone, which he said "acts like magic." Dr. Young has had a case under treatment—the patient, a child eight years old, presenting with a fistulous opening over both deciduous canines, though both were perfectly sound, as were all the other teeth.

The permanent cuspid was plainly perceptible just above the deciduous tooth, and erupted immediately after the extraction of the former. The other deciduous canine was also extracted. Both had live, healthy nerves—there was evidently necrosed bone between the two openings; syringed water, and the probe, passed freely from one to the other, either way.

He was desirous of expression of opinion as to the probable cause of the necrosis and fistulas, all the teeth being sound. The deciduous roots were not absorbed.

Dr. J. Y. Crawford said there is no question but that necrotic action was superinduced by pus burrowing from one point to the other. The primary cause was doubtless the migration of the permanent tooth from a proper to an improper position, irritation from the pressure on the unabsorbed deciduous root causing the inflammation and abscess. Absorption of the roots of the deciduous teeth is an extraordinary though physiological function, and extraordinary function is more susceptible than those which run *pari passu* with existence. Interference with the normal surroundings of a deciduous tooth may cause the arrest of absorption.

Dr. Foster.—Would you expect absorption of the root of a cuspid at that age?

Dr. Crawford.—At nine years of age considerable absorption would not be abnormal. There is no other rational conclusion in this case but that the cause was the migration and premature eruption of the permanent cuspid, traumatic action resulting in the sinus.

Dr. E. S. Chisholm gave the history of a very interesting case. The patient was a child of two years of age, of so decided hemorrhagic diathesis that the slightest scratch was a source of grave anxiety. In running about with a pencil in her mouth the child fell, lacerating the roof of the mouth. For four days every effort had been made by two prominent physicians to arrest the hemorrhage, even to applying the galvanic cautery. They gave the child up to die, as it was vomiting blood, and the system so depleted from loss of blood that they pronounced recovery impossible, even were the hemoarhage checked. Dr. Chisholm was called in by the father to see the child, it occurring to the parents that he so often had occasion to check hemorrhage from the extraction of teeth, etc. He found the child lying in the mother's lap, its death hourly expected. He at once went to work and constructed an appliance of wire to retain a compress in position. He passed a wire around the lateral incisors, throwing out a loop extending beyond the point of laceration, attaching to this a wire passing across from the first molars just erupted, and cutting retaining grooves in the teeth. This served to retain in positing a soft compress of lint, saturated with Monsel's solution—sulphate of iron. The first application stopped the flow of blood, and proved an entire success, the child rallying promptly. On the fifth day it came loose, and while it was being readjusted a physician passed by. The father called to him—being a friend of the family—to come in and see what the dentist had done, and how he had saved the life of their child. But he passed on, saying, "Oh! it would have got well anyhow!"

On another occasion a lady patient brought in her little boy of nine years old, saying that the doctor had ordered all his teeth taken out. The following dialogue ensued:

"I cannot do that."

"Oh! but you must do it; the doctor says it must be done."

"But let me examine his mouth first, before we decide on anything."

"Oh! that will make no difference. You must take them all out, or I shall go somewhere else."

"Very well, you will have to do that; but let me look at his mouth; there does not seem to be much of anything the matter with it. Who is your physician? How long has he been practicing?

"He is a very nice young man who graduated about two years ago."

"Well, Mrs.————, I have been practicing thirty years. My specialty is the teeth, and for twenty-five years I have been studying the teeth of children. Your physician graduated two years ago. He has had no experience in regard to the teeth. Do you place his judgment against mine in such a case as this?"

She laughed, and admitted that she had never looked at it in that light. She simply thought that whatever the doctor said had to be done.

Several cases of so-called cancer cured by proper attention to the teeth were related, the "cancers" proving to be a broken piece of tooth, an abscess from a dead tooth, or from an imbedded wisdom tooth.

A striking case of malpractice was related by Dr. Merril. An old gentleman having an abscess from a decayed wisdom tooth, was treated by two physicians successively for a "boil" on the cheek, hot poultices being applied to the outside and the face lanced on the cheek and under the jaw. The discharge continuing for weeks, he grew seriously ill, and being confined to the house he sent for a younger physician who lived nearer by. The latter at once pronounced the wisdom tooth the cause of all the trouble, and ordered him to the dentist to have it removed. At that time he could scarcely open his mouth enough to pass a finger between his front teeth, and was completely run down from lack of nourishment and the drain on his system. He had not been out of the house for ten weeks and it was several months before he recovered his usual health. But the removal of the wisdom tooth cured him.

The subject of

PYORRHEA ALVEOLARIS

was discussed at considerable length. Dr. Temple uses escharotics, especially Robinson's Remedy," but when the teeth are so loose they can be lifted in the socket, he thinks it preferable to remove them, as they are only a source of continual irritation.

Dr. R. C. Young commends the use of pyrozone in the treatment of this disease. When there are accumulations of sanguinary calculus he does not consider it amenable to local treatment.

Dr. R. R. Freeman spoke of the importance of securing the co-operation of the patient, having him rub the swollen gums thoroughly with a corner of the towel wrapped around the finger, and packing in chalk between his teeth at night. Cleanliness is half the battle.

When a patient comes, for the first time, with the mouth in a condition showing utter ignorance of the use of the tooth-brush, and you feel that you do not want to operate on that mouth for any fee, yet have to be suave and smiling, give him instructions for thoroughly cleansing the mouth night and morning, and put him off for a week, and you will be astonished at the change in conditions that you will not recognize the mouth, but will have to look in your book for the object of the appointment.

Dr. Freeman retains very loose teeth in position by ligating them with fine binding wire to the firmer adjacent teeth, passing the wire in and out and around the teeth till they are held firmly in position. They can thus be rendered serviceable for years.

Dr. Turney gave the treatment of a case which he had finally abandoned as hopeless, nothing availing to arrest the suppuration.

Dr. W. G. Browne has had most favorable results in two recent cases, in which, the patients being traveling men, he had, after thorough removal of all deposits, furnished each with a syringe and vial of peroxid of hydrogen with instructions for use to insure thorough cleanliness. After absence varying from thirty days in one case to sixty in the other, he had been thoroughly astonished at the favorable results.

Dr. J. Y. Crawford emphasized the statement that we don't cure disease; we bind up the parts, and nature works the cure. This is strikingly illustrated in the so-called pyorrhea alveolaris. The tissues involved are not inclined to reproduce themselves; the gum tissue is semi-cartilaginous, and does not respond to treatment; the alveolar process does not reproduce itself, especially in patients who have reached maturity. Seek after palliative treatment. You will get along better if you do not force your surgery too far at first. If salivary and sanguinary calculus have not done their work too thoroughly, you may be able to assist nature by eliminating causes, and mechanically tighten the teeth. You may even cure isolated spots, but we must work on the expectant plan—no stereotyped mode of procedure will answer; we must meet indications as they arise; mitigate conditions. It is a self-limited disease if the patient has vitality to withstand the onslaught. It is a law, that universally holds good, that we can cure nothing but that which has a tendency to cure itself—is self-limited. This gives us new hope, as we better understand constitutional conditions, that we may be able to afford relief through proper treatment. It is not *per se* local; there is an aggravated condition of the secretions from constitutional impression. As to the cause, there is a long list of influences and factors which lead up to and produce it, which we do not understand. I believe that a most potent factor is mal-occlusion of the teeth. Another most potent factor dates away back in the history of the individual. Suppose a child whose baby molars decay at three, four, five years of age. The surroundings are in a state of violent inflammation, while the permanent teeth are in the stage of upbuilding and growth. By virtue of long-continued inflammation, the bone around the permanent teeth is destroyed by necrosis, resulting in a disarrangement of the teeth. In the healing of the soft tissues cicatrical tissue forms, interfering with the eruption of the permanent teeth, with consequent irregularity, mal-occlusion, and pyorrhea alveolaris.

Dr. C. L. Boyd believes that the disease is purely local, neither mal-position nor mal-occlusion being concerned in its production; that it is due to lack of cleanliness and lack of proper exercise of the surrounding tissues, encouraging them to grow out of the jaw a sufficient length to give them space. The gums around the teeth should be rubbed thoroughly twice a day, and children early taught this habit, thus stimulating the tissues and developing the maxillary, so as to give the upper teeth ample room, allowing the lower teeth to go inside without being crowded and jammed together. Exercise is as necessary for the gums and the bony tissues around the teeth as for other portions of the body. Give children such food as requires exercise in mastication. This will develop the jaw and make the teeth healthy and strong; enforce strict cleanliness of the mouth and teeth. In this way only can we avoid the direct causes of decay and pyorrhea alveolaris.

Dr. Crawford said that the ideal mode of cleaning the mouth and teeth would be by a hose attached to the hydrant, throwing water with considerable force. This would be especially good for crown- and bridge-work. Have the water as pure and clean as for baptism.

Dr. R. C. Young.—The worst cases of pyorrhea alveolaris are those where there are no deposits of calculus. It is never cured by the dentist. It sometimes gets well, but not till after the teeth are lost. It is a local manifestation of systemic disease. After the socket has been lost, it is not reproduced any more than an arm. In genuine pyorrhea alveolaris, the roots of the teeth are more than usually polished and glistening, but they are all loose, with pus oozing from around them. The disease is contagious, and may be transferred by the dentist's instruments. I believe that chewing gum, indulged in the privacy of the bed-chamber, as is the case with cleaning the teeth, gives beneficial exercise to the gums and teeth.

Dr. E. S. Chisholm agreed with Dr. Young that calculus is not a necessary feature of the disease, which he defines as a local expression of constitutional disease or vice. We can mitigate it, hold it in check; but we cannot cure it.

Dr. Boyd thinks that neglect of cleanliness, permitting accumulations of tartar on the teeth, would tend to develop constitutional tendency to the disease, and that, therefore, practically, it is due to accumulations of calculus, the development of the tendency being prevented by avoiding known irritants.

Dr. Wm. Crenshaw said that in his own experience he had not found mal-occlusion a factor in producing pyorrhea; on the contrary, some of the worst cases he had known had the teeth in perfect occlusion. His own teeth, for instance, and his father's, were regular, yet both are victims of pericementitis. As a mouth wash, he uses bichloride of mercury, 1 to 1,000, or 4 grains to the fluid pound. He considers this perfectly safe—that a teaspoonful can be swallowed with impunity. This solution of bichlorid of mercury used on the tooth-brush will kill the tendency to pyorrhea alveolaris. Absolute cleanliness is the only treatment of any avail. Go into the pockets and break them up thoroughly, and rinse them out with a strong germicide. The teeth will be stronger and firmer, for awhile at least. Whether a permanent cure can be effected, he was not prepared to say. It is a dread disease attacking the strongest and best teeth, in the strongest constitutions, causing the loss of the soundest teeth.

Dr. Chisholm is satisfied that the disease is hereditary. The deposit in true pyorrhea is at the end of the root, a secretion from the devitalized tissues around the apex, though this is not an unvarying or essential feature.

Dr. L. G. Noel, Nashville, Tenn., regards lowered vitality as the most potent factor in the production of this disease. There is no cure where there is an impoverished condition of the blood, except by the removal of the teeth; but by taking out the first one affected, we can retard its development. In the treatment of pus pockets, after the surgical operation, use Lugol's solution of iodin, peroxid of hydrogen and bichlorid of mercury.

Dr. Crawford criticised Dr. Crenshaw's statement regarding the internal use of bichlorid of mercury, in the strength given—1 in 1,600—saying, "surgeons never use it stronger than 1 to 3,000 in wounds." He thought that if it was so positively hereditary, as claimed, that we would see it in children with the deciduous teeth. But there is never mal-occlusion there, and never pyorrhea alveolaris.

He said that he had reached his conclusions after twenty-five years of close observation. He took exception to the statement that children do not have tartar on the teeth; they do have soft, cheesy tartar, and they have soft chalky teeth that decay early, and are lost prematurely, resulting in distorted, diseased permanent teeth, with mal-occlusion and pyorrhea alveolaris. Disease is not inherited, but a susceptibility, or tendency, to it—it is called a discrasia of the blood.

Dr. R. R. Freeman, Nashville, Tenn., said he thought that something more than irrigation with clear water was necessary to insure cleanliness of the oral cavity. Mechanical manipulation and soap are as necessary here as to the washerwoman, or in cleansing the hands. Even if it be true that we cannot cure pyorrhea, we can use palliative measures, carrying them along, by care and cleansing, in comparative comfort for many years, perhaps. Don't take the teeth out, give them a chance. There is some relief in the mere hope that they may get better. Do not destroy the teeth, except as the very last resort.

Dr. C. L. Boyd said that his teeth offered a striking example of mal occlusion; but that he has no accumulation of tartar, and no predisposition to pyorrhea had as yet made itself apparent.

Dr. W. G. Browne gave an instance of apparent cure of a very clear, typical case of pyorrhea, with no tartar. The tooth affected was a lower incisor. It was treated eight years ago, and remains in position to-day clearly cured.

The molars in the same mouth have since been attacked, but there has been no recurrence of the disease in the tooth treated eight years ago. It was a case of extreme looseness with exudation of pus.

Dr. L. G. Noel.—Pyorrhea alveolaris is a disease of adolescence, pointing to lowered vitality. Children do not have it, because, when the alveolar process is forming, the bones are in active life; there is a high vascularity of the tissues; rich blood is being poured in. Pyorrhea begins after the system has entered on its downward career; many cases occur just before death, whatever the disease.—*Items of Interest.*

